# Professional Development Track to Prepare Future Academic Clinicians

**DOI:** 10.1007/s40670-020-01118-5

**Published:** 2020-10-20

**Authors:** K. Marie Traylor, Jorge L. Cervantes, Cynthia N. Perry

**Affiliations:** Department of Medical Education, Paul L. Foster School of Medicine, Texas Tech University Health Sciences Center El Paso, MSC 21009, 5001 El Paso Drive, El Paso, TX 79905-2827 USA

**Keywords:** Academic medicine, Professional development, Medical education, Distinction program

## Abstract

Professional development is instrumental in the success of professionals and trainees in academic medicine. In response to medical student feedback requesting additional professional development opportunities, the Foster School of Medicine developed a distinction program, the Pathway for Preparing Academic Clinicians (PPAC), designed to deliver sought-after skill development and foundational knowledge in the three primary activities of academic medicine: medical education, research, and patient care. This distinction program addresses a curricular gap as identified by students and common to many UME curricula and also provides an opportunity for residency programs to identify student achievement within a pass/fail program.

**Electronic supplementary material:**

The online version of this article (10.1007/s40670-020-01118-5) contains supplementary material, which is available to authorized users.

## Background

Professional development encompasses the formal and informal learning and training aimed at improving professional performance as it relates to the competencies inherent in medical practice including research, teaching, and patient care [1]. Professional development in medical school is not only important for a health care system aimed to improve patients’ safety and quality of service, but improved understanding of academic medicine career roles may also support development of future academic clinicians [2, 3]. Most undergraduate medical education (UME) programs do not offer formalized professional development content for students interested in an academic career and often extracurricular opportunities are utilized to fill this void [4]. This makes it difficult for medical students to understand the advantages and drawbacks to various career paths including knowing what to expect from a career in academic medicine. Completion of a formal professional development program, focused on academic medicine careers, provides a means for medical students to further distinguish themselves within the residency selection process particularly in the face of evolving changes to UME and may increase the pipeline of future medical school faculty [2, 3].

Although Texas Tech University Health Sciences Center El Paso (TTUHSC El Paso) provides faculty professional development in the areas of clinical skills, teaching, and research through the Office for Faculty Development, medical students at the Paul L Foster School of Medicine (PLFSOM) do not have access to a similar formal curriculum. Rather, student interest groups provide students with extracurricular professional development opportunities which expose students to various specialties and career tracks. Professional development opportunities for students are also presented in Masters Colloquium, one of the core courses within the PLFSOM UME curriculum, although these experiences are limited in scope and frequency. Given these limitations, we speculated that the current formal UME curriculum may leave gaps in professional development of our students as it pertains to non-technical career preparation for entering into the medical profession. In order to assess these possible gaps, we developed a needs assessment survey and explored students’ expressed learning needs to identify deficiencies in our current educational program [5].

## Activity

We developed a needs assessment survey in order to gather pertinent information regarding existing gaps in our curriculum with respect to professional development and career readiness of our students. The survey contained 15 Likert-type scale survey items and 2 open-ended questions, along with demographic information including ethnicity, gender, and year of education (Supplemental Material). The Likert-type scale items had 4 response levels requiring respondents to specify their level of satisfaction or level of agreement with a certain concept. In order to gauge the possible career areas that students were considering entering into, students were asked to select all that applied from the choices of private practice, hospital-based practice, academia, public health, or other. We selected the survey items from previously published surveys or we designed and validated items through peer review and examination for scope and clarity of purpose [6].

While constructing the survey, the authors considered the three primary activities of academic medicine and their respective components for evaluation and participant feedback [7, 8]. Survey questions for section entitled “Student satisfaction with Existing Career Development Offerings” were adapted from Zink et al. [6] and are listed in their entirety in Supplementary materials. The items in the section “Student agreement with Career Expectations” were adapted from DeSimone et al. [9]. Open-ended questions were generated by the authors and vetted by a peer-audience for evaluation of purpose, wording, and intent of each survey item and revised as necessary to improve audience understanding and optimize item quality and reliability.

We distributed the survey link via email to PLFSOM classes of 2019, 2020, 2021, and 2022 after obtaining IRB exemption. We accepted responses during a 2-month period (July and August 2019) and analyzed survey results with Qualtrics software. Responses to the Likert-type scale items were converted from ordinal to numerical responses and treated as interval data [10]. In the open-ended questions, students were asked to identify specific skills they believed they needed to develop for their future career and, in a separate question, skills in which they felt underprepared that may be important in their future career. We thematically grouped the open-ended responses based upon the three primary activities of academic medicine: teaching, research, and patient care [7, 8]. The subgroups were as follows: Practice/Clinic/Business Management (patient care), Daily clinical skills (patient care), Communication/Presentations (teaching), Education on specialties (teaching), Teaching, Research, and Mentorship/Networking (research). Excel was used for formation of all graphics.

## Results

**Fig. 1:**
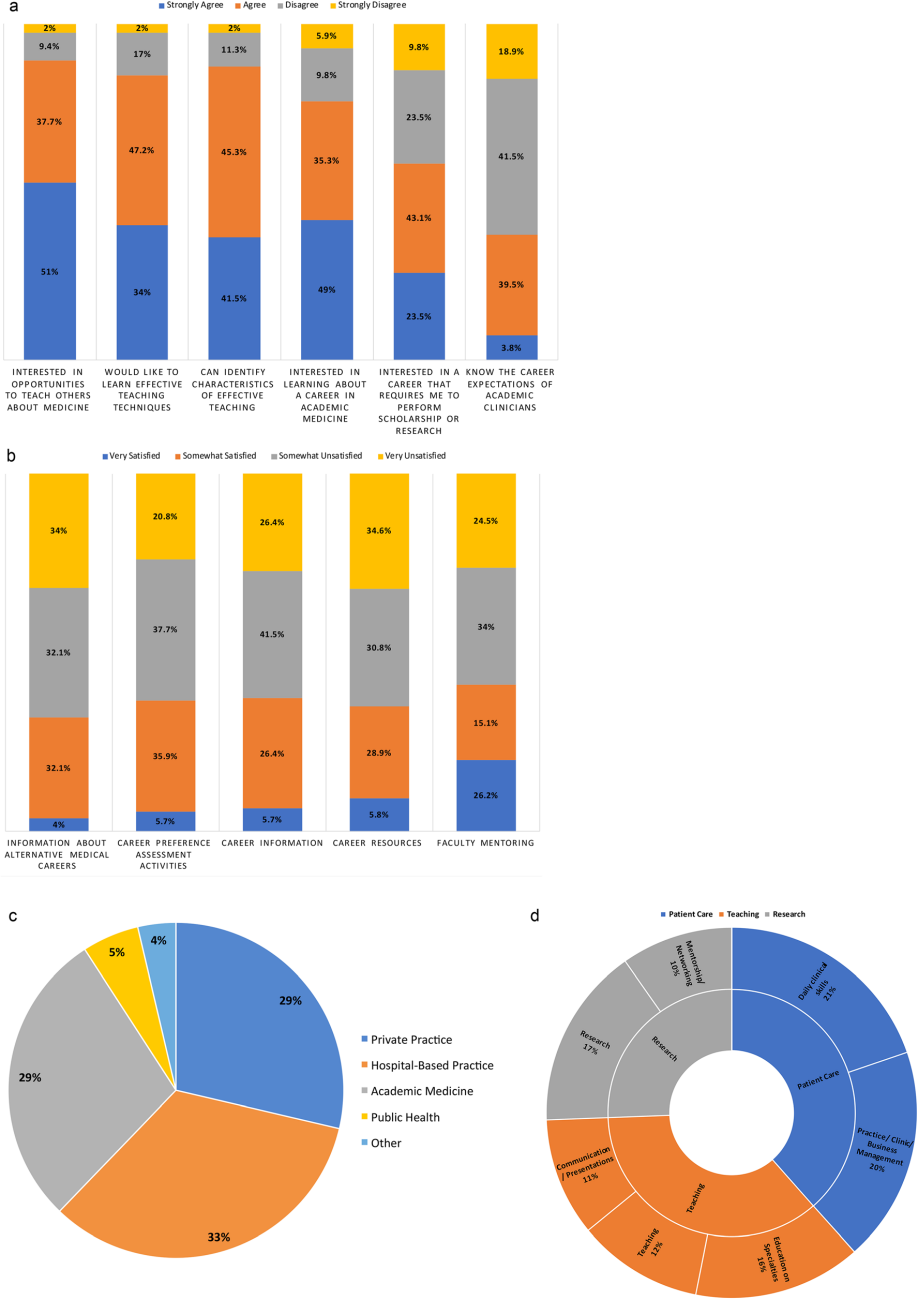
**a** Student agreement with Career Expectations. Survey responses indicate that the majority (> 65%) of students agree they are interested in teaching others, would like to learn more effective teaching techniques, can identify characteristics of effective teaching, are interested in learning more about a career in academic medicine, and are interested in a career that requires scholarship or research. The majority of respondents disagree they know the career expectations of academic clinicians. **b** Student satisfaction with Existing Career Development Offerings. The majority of students are very unsatisfied or somewhat unsatisfied with PLFSOM’s current opportunities in faculty mentoring, career resources, career information, career preference assessment activities, and information about alternative medical careers. **c** Selection of Type of Future Professional Practice. Students selected hospital-based medicine, private practice, and academic medicine as their most likely career choices. **d** Categorized Tally of Responses to Required Skills. Respondents felt the skills required in the future will include daily clinical skills, practice/business/clinic management, research, and education on other specialties

We received 110 completed surveys from the approximately 400 distributed, accounting for a response rate of 27.5%. The results showed that > 65% of respondents agreed or strongly agreed to each of the following statements: I am interested in opportunities to teach others about medicine, I would like to learn effective teaching techniques, I can identify characteristics of effective teaching, I am interested in learning in a career about academic medicine, and I am interested in a career that requires me to perform scholarship or research. On the other hand, students disagreed with the following statement: I know the career expectations of an academic clinician (Fig. 1a).

To identify the main gaps in the existing formal PLFSOM curriculum, we surveyed student satisfaction with provided information regarding alternative medical careers, career preference assessment activities, career information, career resources, and faculty mentoring. The majority of the respondents were either very or somewhat unsatisfied with the current offerings in our curriculum in these areas (Fig. 1b).

When asked to select an intended area of future clinical practice, 33% of students stated they are likely to go into hospital-based practice and 29% of students selected either private practice or academic medicine as the next-most likely career choice (Fig. 1c). When asked to describe what areas students felt underprepared in, business management was the most cited, followed by daily clinical skills, education on specialties, and research (Fig. 1d). These answers were echoed when respondents were asked what skills they need to develop for their future careers. Daily clinical skills (20%), business management (19%), research (15%), and education on other specialties (13%) were the most commonly mentioned skills (Supplemental Materials).

## Discussion

Professional development is instrumental in the success of professionals and trainees in academic medicine. As learners progress through training, opportunities to better understand career roles and responsibilities need to be offered along with the proper education to succeed in academia [5, 8, 11, 12]. Overall, our findings show that there are gaps in our current curriculum most notably related to the three primary activities of academic medicine—medical education, research, and patient care—and their synergy [7, 8]. At the same time, these results also highlight our theory that there is an interest in academic medicine across our medical student body.

In the face of forthcoming changes to USMLE Step 1 scoring and the expansion of pass/fail grading systems at the UME level, medical students and their training programs must identify new means of recognizing student accomplishment. Identification of curricular gaps provides a chance to create novel student learning opportunities that address this challenge while also fulfilling identified curricular deficiencies [13]. To rise to this challenge, we designed a distinction program, the Pathway for Preparing Academic Clinicians (PPAC), which is on trend with nationwide actions in UME programs aiming to provide medical students with targeted career planning and professional development. Our distinction track addresses medical students’ interests as identified in our survey outcomes, providing them with foundational knowledge in adult pedagogy and preparing them for a successful career in academia by offering formalized training in scholarship and teaching, two of the three primary activities of academic medicine (Table 1).

**Table 1:** Pathway for Preparing Academic Clinicians (PPAC) design

**Program Requirements:**	
Participants will be required to attend a Program Orientation, complete a Scholarly Activity and Research Program (SARP) Project in the area of Medical Education Scholarship, complete a 1 year term of Academic Service, fulfill a minimum of 50 hours of career development workshops and prepare and deliver a Capstone Presentation.	
**Sample Workshop Topics (Completion of 50 hours)**	
Why academia?	Assessment in the classroom and bedside
Negotiating an academic contract	Ins and Outs of Team Based Learning
What is academic service and why do I need it?	Securing grant funding 101
Adult Learning and Effective teaching Part I: Theory Part II: Putting it into practice	How to publish from your office Part I: Meta Analysis Part II: Educational Research
Transitioning from Fellow to Faculty	Crafting a submission to MedEd Portal
Institutional structure and leadership	What is tenure and promotion?
Preparing and delivering a good lecture	Art of writing

Implementation of this program will primarily rely on faculty-led instructional workshops but will also piggyback on resources provided by existing campus organizations (e.g., Women in Medicine and Sciences, Faculty Development Program) in order to minimize program costs while providing exposure to expert-level guest speakers. It will also allow for a bidirectional program support with our existing Scholarly and Research Activity Program (SARP), in terms of mentorship and research. This is particularly important given the fact that mentoring has been identified as crucial to the retention and recruitment of trainees in medical and surgical specialties, as well as to promoting research and academia [14].

To measure the success of this program, we will track student progress as they enter into residency and later career path selection. We expect that graduates of this program will be better equipped to transition into residency and fulfill teaching expectations. We are optimistic that the program will develop a pipeline of academics that will return back to our institution to teach, perform research, and provide exceptional patient care.

## Electronic supplementary material

ESM 1 (DOCX 54327 kb)

## Data Availability

NA
