# Supplementary material for: Professional Development Track to Prepare Future Academic Clinicians
Source: Med Sci Educ. 2020 Oct 20;31(1):23–7. doi: 10.1007/s40670-020-01118-5 (PMC8368474; doi:10.1007/s40670-020-01118-5)
Supplement: Supplementary file 1 — (DOCX 54327 kb) [file 40670_2020_1118_MOESM1_ESM.docx]

Supplemental Material:

Survey Questions:

Rate how well you agree with the following statements:

1. The medical school preclinical curriculum prepares me to teach others
2. I am interested in opportunities to teach others, including health professions learners, about medicine
3. I would like to learn about effective teaching techniques
4. I am able to identify characteristics of effective teaching
5. I am interested in learning about a career in academic medicine
6. I am interested in a career that requires me to perform scholarship or research
7. I know the career expectations of academic clinicians
8. I know the career expectations of private clinicians
9. I know the career expectations of public clinicians
10. I can identify career expectations that are important to me

How satisfied with PLFSOM’s

1. Information about alternative medical careers
2. Career preference assessment activities
3. Career information
4. Career resources
5. Faculty mentoring

Which of the following would you most likely enter into as a career? (Select all that apply)

1. Private Practice
2. Hospital-Based Practice
3. Academic Medicine
4. Public Health
5. Other (Please explain)

What skills do you need to develop for your future career? (Open Ended)

What skills do you feel underprepared in that may be needed for your future career?

Please select one:

1. Female
2. Male
3. Other (please specify)

Please select one:

1. MSI
2. MSII
3. MSIII
4. MSIV

I identify my ethnicity as (select all that apply):

1. Asian
2. Black/ African
3. Caucasian
4. Hispanic/ Latinx
5. Native American
6. Pacific Islander
7. Prefer not to answer

**Supplemental Figure 1 Survey Respondant Demograhics** **a)** Ethnicity Self-Identification. Respondent self-reported race identity **b)** Gender Self-Identification. Respondent self-reported gender identity

**Supplemental Figure 2 Grouped Open Ended Student Responses to Areas of Under-Preparation:** Categorized Tally of Responses to Skill Under-Preparation. Respondents feel most underprepared in practice/ business/ clinic management, daily clinical skills, education on other specialties and research.
